# Gamma Knife Surgery for Brain Metastasis from Hepatocellular Carcinoma

**DOI:** 10.1371/journal.pone.0088317

**Published:** 2014-02-07

**Authors:** Qingsheng Xu, Pan Wu, Yiping Feng, Ke Ye, Ying Tong, Yongqing Zhou

**Affiliations:** Department of Neurosurgery, the First Affiliated Hospital, College of Medicine, Zhejiang University, Hangzhou, Zhejiang Province, P. R. China; The George Washington University, United States of America

## Abstract

**Objectives:**

The authors evaluated the results of Gamma knife surgery (GKS) for the treatment of metastatic brain tumors from hepatocellular carcinoma (HCC).

**Methods and Results:**

The authors conducted a retrospective review of the clinical characteristics and treatment outcomes in 14 patients with metastatic brain tumors from HCC who underwent GKS. Twelve (85.7%) patients were male. The mean age of the patients was 53±12 years. There were totally 22 brain metastases in 14 patients and 8 patients (57.1%) presented with a single brain lesion. Intracranial hemorrhages occurred in 13 (59.1%) of the 22 lesions. The mean KPS score was 81±14 (range 50–100). Eleven (78.6%) patients were classified as RTOG RPA Class 2. The mean tumor volume was 8.16±8.15 cm^3^ (range 0.59–27.0 cm^3^). The mean marginal dose prescribed was 18.7±3.2 Gy (range 10.0–22.0 Gy). The mean number of shots administered was 10±9 (range 1–27). The median overall survival time after GKS was 5.0±0.93 months (95% CI 3.2–6.8). No complications related to the radiosurgical treatment were identified. Multivariate analysis showed that the total volume of brain metastases, the RTOG RPA class and serum AFP level were significantly correlated with patients’ survival time.

**Conclusions:**

Although survival was extremely poor in patients with brain metastasis (BM) from HCC, GKS was shown to lead to prolongation of the survival time. Accordingly, GKS can be considered as a valuable treatment option for proper patients with HCC BM.

## Introduction

Hepatocellular carcinoma (HCC) is one of the most common cancers in the world. Better chemotherapy options, aggressive surgery, and liver transplantation have led to improved patients’ survival and an increase in late-appearing, distant metastasis from HCC. Brain metastasis (BM) is being increasingly documented in areas of the world with high endemic rates such as Taiwan, Korea and Japan [1]–[3]. The frequency of BM in patients with HCC reported in earlier studies ranges from 0.2% to as high as 2.2% [2], [4]–[6].

Whole brain radiotherapy (WBRT) has been a standard and efficacious treatment in the management of BM from HCC, but those patients’ outcomes are poor. Multi-modality approaches including surgery and radiosurgery have been investigated to improve survival. However, patients with BM from HCC are often inoperable because of the liver dysfunction and associated coagulopathy. Of note, in recent years, stereotactic radiosurgery, which involves delivering a high dose of radiation into brain lesions in a single session, is also an effective and safe alternative to conventional surgery in patients with intracranial lesions, especially for those with medical comorbidities [7]–[9].

Thus we performed this study to investigate the possible role of GKS in the management of patients with BM from HCC and identify prognostic factors that influence survival time.

## Materials and Methods

### Ethics Statement

This study was approved by the Ethics Committee of the First Affiliated Hospital, College of Medicine, Zhejiang University. And written informed consent was obtained from participants for their clinical records to be used in this study.

### Patients and Setting

The charts of patients with BM from HCC treated by GKS at the authors' institution between August 2011 and April 2013 were retrospectively reviewed.

### Gamma Knife Surgery Protocol

All treatments were performed using the Leksell Gamma knife (Elekta AB) model Perfexion. On the day of treatment, the Leksell frame G was applied to the patient’s head under local anesthesia. A high resolution and thin-slice volumetric gadolinium-enhanced MRI scan was obtained before treatment. Using the GammaPlan (Elekta AB) software, the neurosurgeon, and medical physicists designed the dose plan. The radiosurgery isodose and marginal dose prescribed were usually determined based on the Radiation Therapy Oncology Group (RTOG) 90-05 dosing guidelines with modification [9]. The treatments were usually designed to deliver 50% of the maximal dose to the margins of the target in a single fraction. The final prescription dose expressed as a marginal dose, number of shots which is number of isocenters used in the planning of GKS and other associated treatment parameters were summarized in Table 1. All patients were followed-up, with the first MRI approximately 6 weeks after GKS, then at 3-month intervals.

**Table 1:** Patient characteristics and radiosurgical parameters.

Characteristics	Value
Age (years)	
Mean (mean ± SD)	53±12
Range	27 72
Male: Female	12 (85.7%) : 2 (14.3%)
Cause of liver cirrhosis	
Hepatitis B virus infection	12 (85.7%)
Alcohol	1 (7.14%)
Hepatitis non-B, non-C	1 (7.14%)
AFP^a^ (>400 ng/mL)	9 (64.3%)
Number of lesions	
Single	8(57.1%)
Multiple	6(42.9%)
Presenting symptoms	
Weakness	5 (35.7%)
Headache only	3 (21.4%)
Other	6 (42.9%)
Presence of extracranial metastases	10 (71.4%)
lung	8 (57.1%)
bone	5 (35.7%)
both	3 (21.4%)
none	4 (28.6%)
Karnofsky performance status (KPS) score	
Mean	81±14
KPS≥70	11 (78.6%)
RTOG RPA class^b^	11 (78.6%)
Presence of a hemorrhagic component	13 (59.1%)
Tumor size in volume (cm^3^)	
Mean	8.16±8.15
Median	4.40
Range	0.59 27.0
Marginal does prescribed (Gy)	
Mean	18.7±3.2
Median	20.0
Range	10.0 22.0
Mean number of shots	10±9

a Alpha-fetoprotein;

b The Radiation Therapy Oncology Group recursive partitioning analysis.

### Statistical Analysis

Survival analysis was performed using SPSS Statistics, version 20.0 (SPSS, IBM Corp.). Kaplan-Meier survival plots were used to demonstrate the overall survival time and progression-free survival rate. The log-rank test (significance level, α = 0.05) was used to assess differences in the overall survival distributions between groups. Cox proportional hazards regression analyses (significance level, α = 0.05) were used to identify independent variables categorically associated with patients or lesions, as well as to adjust for covariates, and the backward stepwise variable selection method was used to control for multicollinearity among the covariates. Variables include age (65 years as cutoff value), gender (female vs. Male), the alpha fetoprotein (AFP) level (400 ng/mL as cutoff value), Karnofsky performance status (KPS) score (70 as cutoff value), RTOG recursive partitioning analysis (RPA) Class (Class 3 vs. Class 2), number of BM (single vs. multiple) and total volume of brain metastases (14 cm^3^ as cutoff value).

## Results

### Patient Characteristics

A total of 14 patients were diagnosed with BM from HCC, of which 12 (85.7%) patients were male. The mean age of the patients was 53±12 years (range 27–72). Hepatitis B virus infection was detected in 12 patients (85.7%). None of the patients was infected with hepatitis C virus. At the time of diagnosis of BM, primary HCC was controlled in 5 patients (35.7%), and the median alpha-fetoprotein (AFP) level was 822 ng/mL (range 1.4–19108.7). Ten patients suffer extracranial metastasis. The most common site of extracranial metastasis was the lung, with 8 (57.1%) patients affected. The median interval between diagnosis of HCC and the development of BM was 26 months (range 2–62). There were totally 22 brain metastases in 14 patients. Eight patients (57.1%) presented with a single brain lesion, and brain lesions were found in the frontal lobe, the parietal lobe, the occipital lobe and brain stem. And intracranial hemorrhages occurred in 13 (59.1%) of the 22 lesions. The most common presenting symptoms were motor weakness (35.7%) and headache (21.4%). The mean KPS score was 81±14 (range 50–100). Eleven (78.6%) patients were classified as RTOG RPA Class 2, and three (21.4%) patients were classified as Class 3.

### Treatment

One patient had been treated with surgical resection for BM that had caused intracerebral hemorrhage with a profound mass effect, and GKS was performed on the residual tumors. GKS was the primary treatment for BM in the remaining 13 (59.1%) patients. The mean tumor volume was 8.16±8.15 cm^3^ (range 0.59–27.0). The mean marginal dose prescribed was 18.7±3.2 Gy (range 10.0–22.0). The mean number of shots administered was 10±9 (range 1–27).

### Outcome and Prognostic Analysis

**Figure 1:**
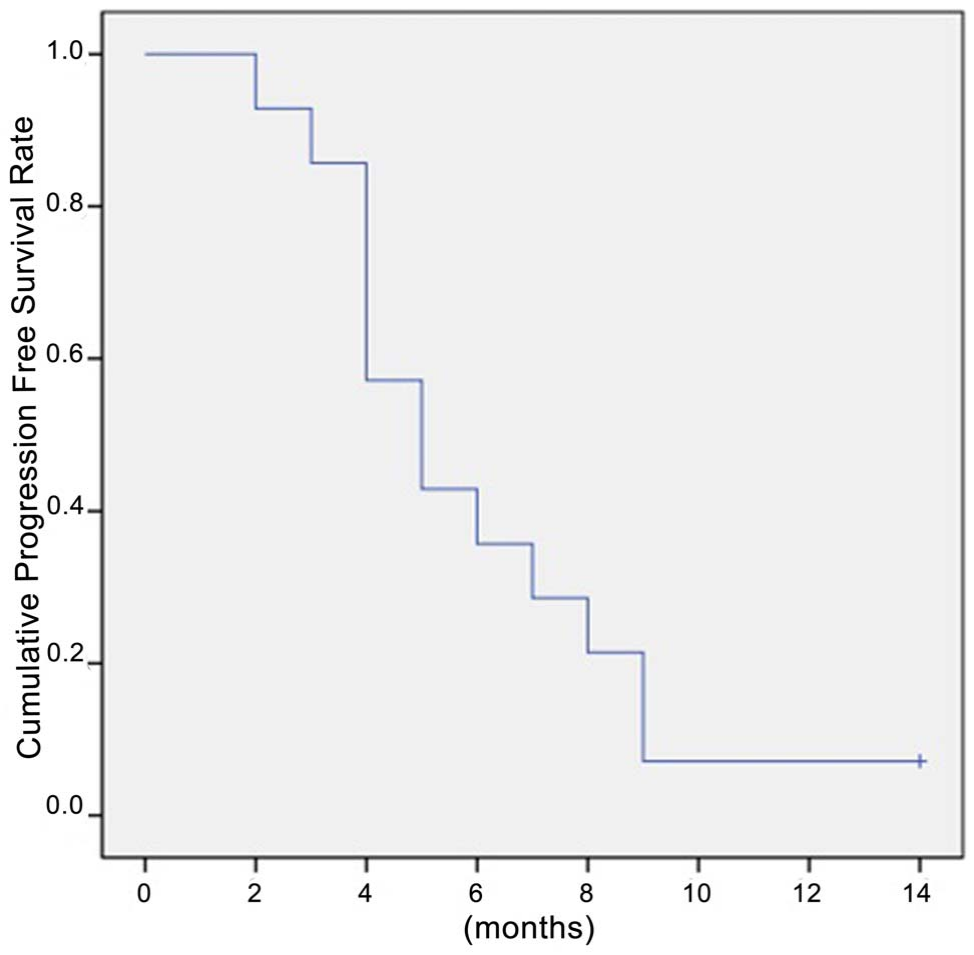
Kaplan-Meier estimates of overall survival. The median overall survival time after GKS was 5.0±0.93 months (95% CI 3.2–6.8). The actuarial survival rates were 92.9, 85.7, 57.1, 42.9, 35.7, and 28.6% at 2, 3, 4, 5, 6 and 7 months after GKS, respectively.

**Figure 2:**
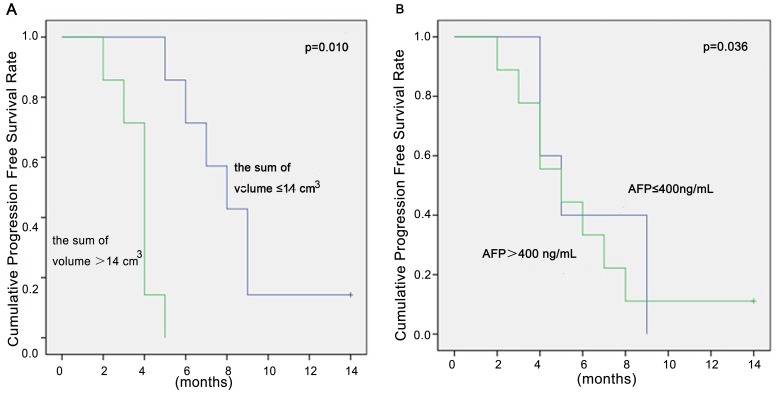
Survival estimates based on the total volume of brain metastases and the alpha-fetoprotein level. (A) Estimates differed significantly for volumes of brain metastases >14 cm3 using the Cox proportional hazards model with backward stepwise variable selection (HR = 37.461; 95% CI 2.381–589.472; p = 0.010). (B) Estimates differed significantly for AFP levels >400 ng/mL using the Cox proportional hazards model with backward stepwise variable selection (HR = 6.223; 95% CI 1.131–34.236; p = 0.036).

Among 13 of the 14 patients who died during the follow-up period, 12 (85.7%) succumbed to systemic disease progression and 1 (7.14%) neurological death as determined using the protocol described by Patchell et al [10]. One (7.14%) patient was alive at the end of follow-up. The median overall survival time after GKS was 5.0±0.93 months (95% CI 3.2–6.8) (Figure 1). Results of variables analyzed for overall survival were shown in Table 2. Total volume of brain metastases ≤14 cm^3^ and AFP levels ≤400 ng/mL were positively associated with survival time for patients of BM from HCC (Figure 2). No complications related to the radiosurgical treatment were identified.

**Table 2:** The results of statistical analyses for overall survival.

	Overall survival				
	Univariate		Multivariate		
	HR	*p value*	HR	*p value*	95% CI
Age (>65 years)	0.419	0.411		0.149	
Female gender	0.348	0.198		0.821	
AFP^a^ (>400 ng/mL)	1.273	0.679	6.223	0.036	1.131 34.236
Karnofsky performance status (>70)	8.997	0.009		not performed	
RTOG RPA class 3 versus 2^b^	8.997	0.009	8.108	0.047	1.030 63.796
Single versus multiple	0.711	0.558		0.689	
Total tumor volume (>14 cm^3^)	17.148	0.011	37.461	0.010	2.381 589.472

a Alpha-fetoprotein;

b The Radiation Therapy Oncology Group recursive partitioning analysis.

## Discussion

BM from HCC is the terminal clinical event in the disease’s course. WBRT has been a standard and efficacious treatment in the management of BM, but the outcomes remain dismal. Multimodality approaches including surgery, radiosurgery, and WBRT have been investigated to improve survival. The median survival for patients with BM from HCC was from 4 to 12 weeks [1], [3], [11]. Our institutional experience with Gamma knife treatment of BM from HCC showed considerably prolonged survival (5.0±0.93 months). The possible explanation may be the current patients have high KPS score and lower RPA class. According to the literature, RPA class has been consistently associated with an impact on survival [12]–[13]. In this study, we determined that lower RPA class was a positive predictor of response to GKS for BM from HCC and the RPA classes 2 was suggestive of better survival. This finding suggests that RPA classification may be useful as a predictor of survival for HCC patients with BM. Serum AFP was the most useful tumor-marker reflecting tumor burden in HCC [14]–[15]. Accordingly, it seems logical that lower serum AFP levels (≤400 ng/ml) was positively associated with survival. On multivariate analysis, lower serum AFP level was one of the most powerful prognostic factors for favorable survival (HR = 6.223; 95% CI, 1.131–34.236; p = 0.036).

Furthermore, we determined that age, gender and the number of brain metastatic lesions were not prognostic factors for improved survival.

In accordance with the RTOG 90-05 dosing guidelines [16], we prescribed ≥20 Gy for lesions with volumes ≤14 cm^3^, and ≤18 Gy for lesions with volumes of >14 cm^3^. We found that the marginal dose prescribed of more than 20 Gy appears to be sufficient to control the lesions in the brain due to HCC. Hiraoka et al [17] also reported that they were able to control BM from HCC and preserve neurological function using a marginal dose of 35 Gy in 5 fractionations(biologically effective dose approximately 20 Gy in a single fraction). Han JH et al [13] reported that prescribed dose less than 20 Gy appears to be insufficient to control the lesions, resulting in poor survival outcomes for patients with tumor volumes of 14 cm^3^ or more. Our finding that smaller tumor volume (≤14 cm^3^) was positive associated with survival time also supports this claim.

Another reason for the poor prognosis in patients with BM from HCC may be due to the characteristic tendency to bleed. In the present study, approximately 59.1% of lesions demonstrated a hemorrhage, compared to 46.3%–54.8% recorded in previous reports [1], [3], [11], [18]. The presence of a hemorrhagic component can interfere with GKS. A signal change on MR imaging and blurring of the lesions due to tumor bleeding often makes radiosurgery planning difficult [19]. In addition, intratumoral hemorrhage often increases the volume of tumor, which results in a relatively low marginal dose being prescribed. This in turn leads to failure of local control [13].

The present study had several limitations. The clinical features of the patients were reviewed retrospectively. Our patient sample size was relatively small and the patient population was heterogeneous in terms of systemic comorbidities. However, considering the rarity of the disease, this study can provide useful information on the management of BM from HCC.

## Conclusions

In summary, although survival was extremely poor in patients with BM from HCC, GKS was shown to lead to prolongation of the survival time, especially in those with lower RPA class and serum AFP level. Accordingly, GKS can be considered as a valuable treatment option for proper patients with HCC Brain Metastasis.
